# Intracranial hypotension secondary to spinal arachnoid cyst rupture presenting with acute severe headache: a case report

**DOI:** 10.1186/1752-1947-4-406

**Published:** 2010-12-17

**Authors:** Wendy D Jones, Makarand Kulkarni, K Ravishankar, Rudi Borgstein, Peter Dupont

**Affiliations:** 1 Department of Nephrology and General Medicine, North Middlesex University Hospital, Sterling Way, London, UK; 2 Department of Radiology, Lilavati Hospital and Research Centre, Mumbai, India; 3 The Headache and Migraine Clinic, Lilavati Hospital and Research Centre, Mumbai, India; 4 Department of Radiology, North Middlesex University Hospital, Sterling Way, London, UK

## Abstract

**Introduction:**

Headache is a common presenting complaint and has a wide differential diagnosis. Clinicians need to be alert to clues that may suggest an underlying secondary aetiology. We describe a novel case of headache secondary to intracranial hypotension which was precipitated by the rupture of a spinal arachnoid cyst.

**Case report:**

A 51-year-old Indian female presented with sudden onset severe headache suggestive of a subarachnoid haemorrage. Investigations including a computed tomography brain scan, cerebrospinal fluid examination and a magnetic resonance angiogram were normal. The headache persisted and magnetic resonance imaging revealed bilateral thin subdural collections, a spinal subarachnoid cyst and a right-sided pleural effusion. This was consistent with a diagnosis of headache secondary to intracranial hypotension resulting from spinal arachnoid cyst rupture.

**Conclusions:**

Spinal arachnoid cyst rupture is a rare cause of spontaneous intracranial hypotension. Spontaneous intracranial hypotension is a common yet under-diagnosed heterogeneous condition. It should feature significantly in the differential diagnosis of patients with new-onset daily persistent headache.

## Introduction

Headache is a common presenting complaint. It is the most frequent neurological complaint seen in general practice [1], accounts for up to one-third of new outpatient neurology referrals [2], and is commonly seen in emergency departments [3]. Most cases are due to so-called primary headache. However, clinicians need to be alert to clues, which may help to identify the minority with an underlying secondary cause. The differential diagnosis of acute severe headache covers a wide range of conditions, including subarachnoid haemorrhage, bacterial meningitis and migraine. We present a novel case of headache secondary to spontaneous intracranial hypotension, which was precipitated by the rupture of a spinal arachnoid cyst.

## Case presentation

A 51-year-old Indian female presented to the emergency department of her local hospital with a history of sudden-onset, severe bilateral occipital headache. Her headache had started suddenly three days earlier with no prodromal symptoms or preceding visual aura. The headache was continuous, exacerbated by coughing and relieved when she was recumbent. The pain radiated across the top of her head to the temporal region and was associated with nausea and mild photophobia. There was no relief with simple analgesia. A history of a recent coryzal illness was elicited but that had fully resolved by the time of presentation.

Her past medical history was unremarkable and she was on no regular medications. There was no history of migraine and no family history of headaches, cerebrovascular accident or sudden death.

On examination, the patient was alert and her vital signs were within normal limits. Neurological examination was normal and Kernig's sign was negative. There was no neck stiffness and no rash. Fundoscopy showed no signs of raised intracranial pressure.

Initial investigations revealed a mild leucocytosis with a neutrophilia but inflammatory markers were normal (erythrocyte sedimentation rate 32 mm/hour; C-reactive protein level <5 mg/L).

Her history of acute-onset severe headache suggested a diagnosis of subarachnoid haemorrhage. A contrast-enhanced computed tomography (CT) brain scan proved normal. A lumbar puncture showed no evidence of red cells in the cerebrospinal fluid (CSF) and the opening pressure was normal at 11 cm/H_2_O. Spectrophotometric examination of the CSF was negative for xanthochromia. A magnetic resonance angiogram was performed to exclude the presence of an intracranial aneurysm. This was normal.

In light of the negative investigations and symptomatic improvement with bed rest and weak opioid analgesia, a tentative diagnosis of migraine was made and the patient was discharged.

**Figure 1:**
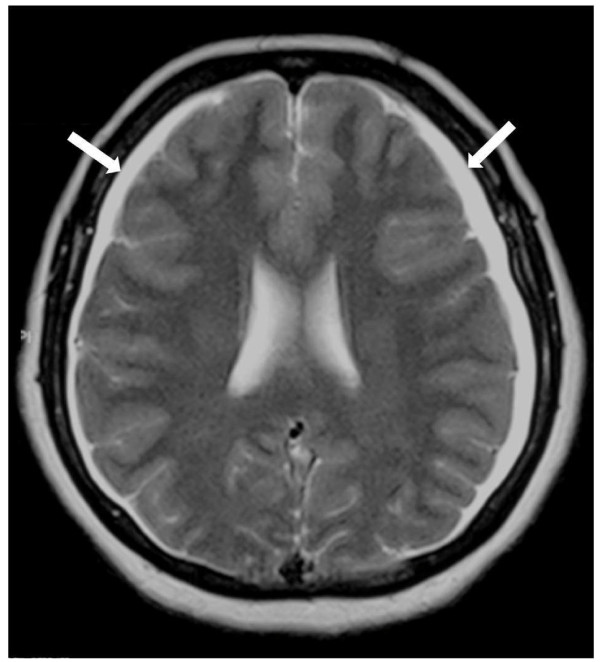
**T1-weighted magnetic resonance scan of the head**. Bilateral thin subdural collections (arrowed).

**Figure 2:**
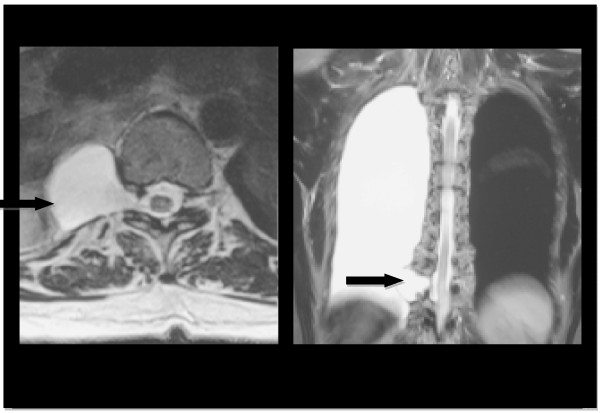
**T2-weighted magnetic resonance scan of the spine showing a large para-spinal arachnoid cyst (arrowed) at the level of T10/11**. This extends out through the right-sided neural foramen. The pleural effusion on the right suggests cyst rupture.

Two months later, although symptomatically improved, the patient still had persistent headache and sought a further opinion. A repeat magnetic resonance imaging (MRI) brain scan revealed new bilateral thin (<1 cm) subdural collections suggesting the possibility of reduced intracranial pressure (Figure 1). An MRI of the spinal cord demonstrated a large right-sided spinal arachnoid cyst at the level of T10/11 extending out through the neural foramen (Figure 2). A right-sided pleural effusion was also noted suggesting that the cyst had ruptured into the pleural space.

In retrospect it seems likely that the patient's initial presentation was precipitated by spontaneous rupture of the spinal arachnoid cyst. Her persistent headache was the result of ongoing CSF hypotension.

Twelve months after initial presentation our patient is well and her headache has completely abated. Her right-sided pleural effusion has resolved and she has not required any neurosurgical intervention.

## Discussion

Spontaneous intracranial hypotension has been recently recognised as a significant and under-diagnosed secondary cause of headache [4]. An epidemiological study suggests that it has an incidence approaching that of subarachnoid haemorrhage [5], yet initial misdiagnosis remains the norm. Classically the headache is orthostatic, worsening when the affected person assumes an upright position and improving when the person lies down. While this pattern is well recognised when associated with a CSF leak following lumbar puncture, the spontaneous onset of orthostatic headache is not, and patients are often initially mislabelled as having migraine, tension headache or some other cause.

Most cases of spontaneous intracranial hypotension are not due to dramatic rupture of arachnoid cysts but rather due to the spontaneous slow leakage of CSF via small dural defects. The precise mechanism by which these dural defects arise is unknown but they are thought to represent an underlying meningeal weakness [6]. There is evidence for a generalized connective tissue disorder in up to two-thirds of patients [7].

The diagnosis of spontaneous intracranial hypotension is made mainly on clinical grounds once other causes of headache have been excluded. Orthostatic headache is typical, but all types of headache have been reported [4]. Other symptoms reported include posterior neck pain or stiffness, nausea and vomiting and photophobia [4,6]. Rarely, patients may present with a decreased level of consciousness due to severe brain displacement [4].

The diagnosis is most often confirmed by cranial MRI scanning where the characteristic features are subdural fluid collections (seen in 50% of cases) [8], pachymeningeal enhancement, engorgement of venous structures, pituitary hyperaemia and sagging of the brain [4]. A recent study suggested spinal MRI is useful, especially in the early stages [9].

On lumbar puncture, opening pressure is typically less than 60 mm H_2_O (Reference range 65 to 195 mmH_2_O), although a normal opening pressure does not exclude the diagnosis [6]. Myelography may have a role in determining the exact site of the leak [6].

Most cases of spontaneous intracranial hypotension resolve with conservative management [4]. This includes bed rest and increased oral fluid intake. Specific medical therapies suggested include glucocorticoids, intravenous caffeine and theophylline; however, these have limited effectiveness [4]. Surgical treatment is reserved for those in whom non-surgical measures have failed. Surgical options include epidural blood patching [10] and percutaneous placement of a fibrin sealant [4]; however, treatment outcomes have been poorly studied [4].

It is important to consider the pathophysiological changes resulting from intracranial hypotension within the intracranial compartment. According to the Monro-Kellie doctrine, the sum of the volumes of the brain, CSF and intracranial blood is constant, with an increase in the volume of one component causing a decrease in the volume of one or both of the other two [11,12]. This hypothesis would explain many of the MRI abnormalities seen in intracranial hypotension [13], including those seen in our case. These abnormalities include subdural fluid collections, meningeal enhancement, engorgement of cerebral venous sinuses and enlargement of the pituitary gland.

However, there is increasing clinical and experimental evidence to suggest that a more accurate model of intracranial pressure dynamics is one involving different intracranial compartments, each related to different brain regions [14]. Increased understanding of intracranial pressure dynamics is likely to guide the management of spontaneous intracranial hypotension in the future.

## Conclusions

Spinal arachnoid cyst rupture is a rare cause of spontaneous intracranial hypotension. Spontaneous intracranial hypotension is a common, yet under-diagnosed, heterogeneous condition. It should feature significantly in the differential diagnoses of patients with new-onset daily persistent headache. This novel case has highlighted the importance of considering spinal MRI in patients presenting with spontaneous intracranial hypotension.

## Consent

Written informed consent was obtained from the patient for publication of this case report and accompanying images. A copy of the written consent is available for review by the Editor-in-Chief of this journal.

## Competing interests

The authors declare that they have no competing interests.

## Authors' contributions

The paper was conceived by PD. The paper was co-authored by WJ and PD. MK and KR provided the clinical radiology images for the manuscript. RB converted the radiology images to digital format and assisted with interpretation of the findings. All authors were directly involved in the clinical care of the patient and all have reviewed and approved the final manuscript. PD will act as guarantor for the manuscript and is the corresponding author.
